# *Citrus limon* Peel Extract Modulates Redox Enzymes and Induces Cytotoxicity in Human Gastric Cancer Cells

**DOI:** 10.3390/ijms27020598

**Published:** 2026-01-07

**Authors:** Rosarita Nasso, Rosario Rullo, Antonio D’Errico, Pierluigi Reveglia, Lucia Lecce, Annarita Poli, Paola Di Donato, Gaetano Corso, Emmanuele De Vendittis, Rosaria Arcone, Mariorosario Masullo

**Affiliations:** 1 Department of Medical, Movement and Well-Being Sciences, Via Medina, 40, 80133 Napoli, Italy; rosaritanasso@gmail.com (R.N.); antonio.derrico002@studenti.uniparthenope.it (A.D.); rosaria.arcone@uniparthenope.it (R.A.); 2 Institute for the Animal Production Systems in the Mediterranean Environment, National Research Council, Piazzale Enrico Fermi 1, 80055 Portici, Italy; rosario.rullo@cnr.it; 3 Department of Clinical and Experimental Medicine, University of Foggia, Viale Pinto, 1, 71122 Foggia, Italy; pierluigi.reveglia@unifg.it (P.R.); lucia.lecce@unifg.it (L.L.); gaetano.corso@unifg.it (G.C.); 4 Institute of Biomolecular Chemistry, National Research Council, Via Campi Flegrei 34, 80078 Pozzuoli, Italy; annarita.poli@cnr.it; 5 Department of Science and Technologies, University of Naples “Parthenope”, Centro Direzionale Isola C4, 80143 Napoli, Italy; paola.didonato@uniparthenope.it; 6 Department of Molecular Medicine and Medical Biotechnologies, University of Naples Federico II, Via S. Pansini 5, 80131 Napoli, Italy; devendit@unina.it

**Keywords:** *Citrus limon* peel extract, gastric cancer, polyphenols, oxidative stress, ROS modulation

## Abstract

Gastric cancer remains a leading cause of cancer-related mortality worldwide. Citrus fruits are rich in polyphenols, exerting antioxidant and chemo-preventive activities, and lemon peel represents a valuable source of such bioactive compounds. Previous studies showed that *Citrus limon* peel extracts (LPE) inhibited the activity of some enzymes of the antioxidant system and reduced the interleukin-6-dependent invasiveness of gastric and colon cancer cells. In the present study, we have investigated the effects of LPE on the human gastric adenocarcinoma AGS and MKN-28 cells and on the activity of a crucial redox enzyme, catalase (CAT). Indeed, LPE significantly reduced the cell viability and clonogenic potential of the gastric cancer cells and induced morphological changes indicative of cytotoxicity. Moreover, LPE modulated the intracellular redox homeostasis by decreasing levels of the hydrogen peroxide-related reactive oxygen species (ROS) while increasing those of superoxide anions and decreasing levels of superoxide dismutases (SODs). Western blotting analysis revealed that LPE downregulated CAT, SOD-1, SOD-2, and monoamine oxidase A (MAO-A) protein expression level in both cell lines. Finally, the extract inhibited CAT activity in a dose-dependent manner (IC_50_ = 0.008 ± 0.003 mg/mL; *K*_i_ = 0.012 ± 0.002 mg/mL). These findings indicate that LPE exerts cytotoxic and redox-modulating effects through the inhibition of antioxidant enzymes and the alteration of ROS balance. Therefore, the agro-industrial by-product LPE could be considered as a promising natural source of polyphenolic compounds with potential applications in the prevention and therapy of gastric cancer.

## 1. Introduction

Gastric cancer (GC) is the primary epithelial malignancy in the stomach. It is the fourth most common cause of tumor-related mortality and the fifth for incidence in the world [1]; therefore, GC remains a significant global health issue. GC is a multifactorial disease associated with various risk factors, some of which are related to prognosis and survival [2]. Chronic *Helicobacter pylori* infection is considered the principal cause of GC. Other risk factors may include family history, gastrointestinal microbiota, obesity, diet, tobacco and alcohol consumption, chemicals, and radiation or virus exposure [3]. Unhealthy dietary intake and lifestyle factors may account up to 50% of all GC cases. Previous studies have shown that high fruit consumption is associated with decreased GC risk; indeed, an increased intake of fresh fruit and the restriction in salt and salt-preserved food consumption prevent the onset of GC. Fruit, particularly citrus fruit, contains a high concentration of vitamin C, carotenoids, polyphenols, and antioxidants, which protect against oxidative damage. These nutrients may play a protective role in the carcinogenesis process [3,4]. Several reports have demonstrated the antioxidant and chemo-preventive properties of dietary polyphenols in cancer prevention [5,6,7]. In addition to these properties, polyphenols can regulate various signal transduction pathways with multiple molecular targets linked to cell survival, proliferation, differentiation, migration, invasion, inflammation, and angiogenesis [8,9]. In this regard, growing evidence highlights the biomedical importance of flavonoids either as complex matrices from natural extracts [10,11] or as individual components [12,13] for the treatment of human diseases. From the mechanistic point of view, very recent studies have reported the involvement of these compounds in the regulation of autophagy and apoptosis [14,15,16], pathways including redox shift at cellular levels.

The lemon peel extract (LPE) from residues of the industrial processing of lemon (*Citrus limon*) has been recently studied for its anti-cancer properties in various cancer types [17,18]. Citrus peels, which are usually considered waste, contain various bioactive compounds, particularly flavonoids and essential oils [19]. Due to their diverse composition, citrus peels exhibit antioxidant and anti-inflammatory properties, as well as other biological benefits to human health. They may act as chemo-preventive and chemotherapeutic agents, either alone or in conjunction with other drugs [20,21]. In our previous studies, we have demonstrated that LPE reduces the interleukin-6-dependent invasiveness of human gastric adenocarcinoma (AGS and MKN-28) and primary colon (T88 and T93) cancer cells through the STAT-3 signaling pathway [22,23]. In addition, LPE also inhibits the in vitro activity of key enzymes, like acetylcholinesterase (AChE) [22], butyrylcholinesterase (BuChE), monoamine oxidases A/B (MAO-A/B), superoxide dismutases 1/2 (SOD-1/2), and the Aβ1–40 in vitro aggregation [24]. Taken together, all these results suggest that LPE has chemo-preventive and neuroprotective potential [25,26]. The aim of this study was to investigate the novel biological properties of LPE, which could suggest its possible consideration as an anti-cancer agent in human GC cells. The results obtained proved that LPE displayed a cytotoxic effect on AGS and MKN-28 GC cells, was able to reduce cell viability, and modulated the ROS production and protein levels of enzymes involved in oxidative stress in these GC cells. Moreover, LPE inhibits in vitro catalase (CAT) activity.

## 2. Results

### 2.1. Identification of Polyphenols Extracted from Lemon Peel Extract (LPE)

Lemon peel extract (LPE) is a rich source of polyphenols, which have been described as having healthy properties and being effective in the treatment of cancers. The extract was subjected to LC-MS/MS analysis to characterize its constituents, using 81 standard polyphenols, comprising 43 phenolic acids and 38 flavonoids, as previously reported [27]. The results obtained from the Multiple Reaction Monitoring (MRM) analysis are reported in Table 1. Indeed, among ten phenolic acids identified in LPE, the most abundant were ferulic acid, *p*-coumaric acid, trans-2-hydroxycinnamic acid, and 4-hydroxybenzoic, in order; concerning the eleven identified flavonoids, the most abundant were hesperidin, rutin hydrate, myricetin, kaempferol, (+/−) naringenin, and diosmetin, in that order. Other phenolic acids and flavonoids were below the limit of quantification (LOQ) or detection (LOD). These results confirm previous findings by Di Donato et al. [17], with the addition of new identified components.

### 2.2. Effect of LPE on the Morphology of AGS and MKN-28 Gastric Adenocarcinoma Cells

**Figure 1:**
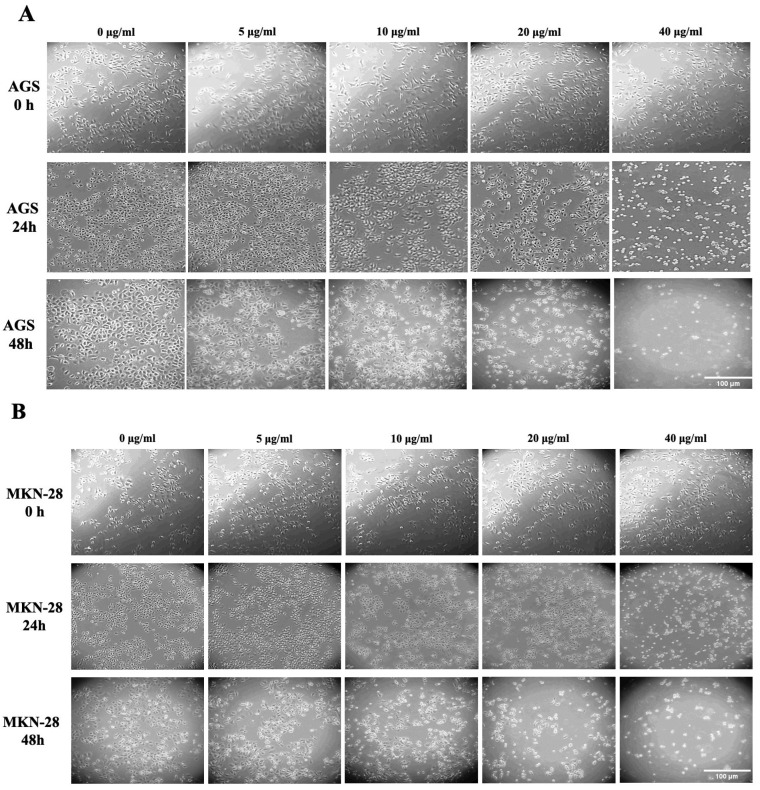
Effect of LPE on the cell morphology of AGS and MKN-28 gastric adenocarcinoma cell lines: (**A**) AGS cells; (**B**) MKN-28 cells were treated with vehicle alone (PBS) or 5, 10, 20, and 40 µg/mL LPE for 24 and 48 h. Images are representative of three independent experiments. Magnification ×10.

AGS and MKN-28 cells were incubated for 24 and 48 h with PBS, as a vehicle alone, or with increasing concentrations of LPE; then, morphological changes were monitored by a phase-contrast light microscopy (Figure 1). After 24 h treatment, morphological changes were evident in both cell lines at the maximum 40 µg/mL LPE concentration. The cells appeared with round cell bodies and with a lower density compared to the control cells, suggesting a detachment from the surface of the tissue culture dish and/or cell death. This effect became more evident after 48 h in cells treated with 20 and 40 µg/mL LPE. These cell morphological alterations can be associated with a toxic action exerted by the extract. To investigate the specific cytotoxic effect exerted by LPE on gastric cancer cells, BJ-5ta cells, a normal immortalized fibroblast cell line, were incubated with the same concentration of the extract for 24 and 48 h (Supplementary Figure S1). Compared with AGS and MKN28 cells, the morphology of fibroblasts does not appear greatly altered by treatment with LPE. Therefore, the absence of significant morphological changes in the normal cell line suggests that LPE is more likely to have a toxic effect in cancer cells than in normal cells.

### 2.3. Effect of LPE on Cell Viability of AGS and MKN-28 Cells

**Figure 2:**
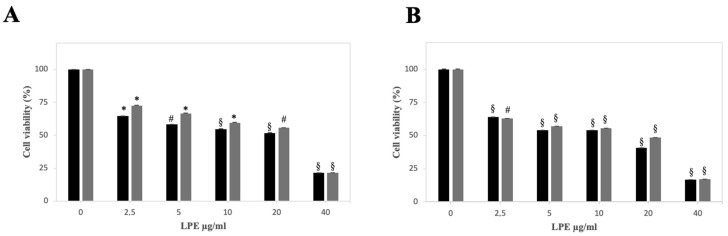
Cell viability of human adenocarcinoma cell lines after treatment with LPE. AGS (black columns) and MKN-28 (grey columns) cells were treated for 24 h (**A**) and 48 h (**B**) with the indicated concentrations of LPE. Control cells were incubated with PBS as a vehicle. Cell viability was determined with the MTT assay, as reported in Materials and Methods. The values, reported as a percentage compared to untreated control cells, represent the mean ± SE of separate experiments performed in triplicate. The significance was evaluated with *p* < 0.05 (*), 0.01 (#), and 0.001 (§).

The toxic effect exerted by LPE on the morphology of AGS and MKN-28 cells was also investigated. In a previous work, we have already assessed the ability of LPE to affect AGS and MKN-28 cell proliferation [22]. To confirm this finding, we have performed an MTT assay to evaluate the cell viability in the presence of different concentrations of the extract. AGS and MKN-28 cells were treated for 24 and 48 h with PBS, as a vehicle alone, or with increasing concentrations of LPE. The data shown in Figure 2 indicate that LPE reduced the cell viability of both gastric adenocarcinoma cell lines in a dose- and time-dependent manner. After 24 h, cell proliferation was progressively reduced, starting from the low concentration of this extract; at the maximum LPE concentration, cell viability was reduced to 21% in both cell lines (Figure 2A) and the calculated IC_50_ value was 22 µg/mL and 21 µg/mL for AGS and MKN-28, respectively. After 48 h of treatment, a similar trend was observed, and at the maximum LPE concentration, cell viability was reduced to 17% in both cell lines (Figure 2B). The IC_50_ value was 18.2 µg/mL and 18.6 µg/mL for AGS and MKN-28, respectively.

### 2.4. Effect of LPE on the Colony Formation Capability of AGS and MKN-28 Cells

**Figure 3:**
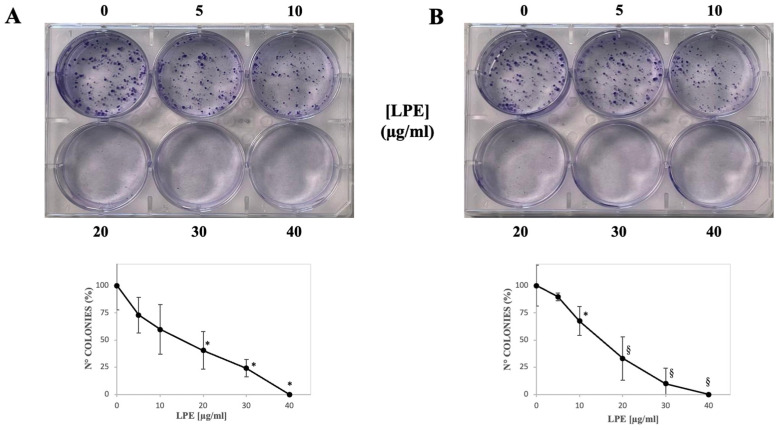
Effect of LPE on the colony-ability of AGS and MKN-28 gastric adenocarcinoma cells. (**A**) AGS cells and (**B**) MKN-28 cells were seeded into six-well culture plates and treated with PBS (vehicle alone) or 5, 10, 20, 30, or 40 µg/mL LPE for 7 days. Then, plates were photographed and images of representative experiments are shown. Plots report the number of colonies counted as reported in the Materials and Methods. The values, reported as a percentage compared to untreated control cells, represent the mean ± SE of separate experiments performed in triplicate. The significance was evaluated with *p* < 0.05 (*) and 0.001 (§).

To further evaluate the long-term cell growth inhibitory effect by LPE, a colony-forming assay was performed. This test is widely used in cancer research, as the capacity to form clones represents a typical trait of cancer cells; furthermore, it represents a standard tool for evaluating the long-term cytotoxic effects of various agents with potential clinical application [28]. Hence, AGS and MKN-28 cells were treated with PBS or increasing concentrations of LPE, and the number of colonies was detected (Figure 3). LPE affected colony formation in a progressive and remarkable manner. Indeed, in both AGS (Figure 3A) and MKN-28 cells (Figure 3B) a significant reduction in the number of colonies was already observed in the presence of low LPE concentrations, i.e., 20 µg/mL for AGS and 10 µg/mL for MKN-28; furthermore, in both cell lines, the maximum dose of LPE completely inhibited the formation of colonies.

### 2.5. Effect of LPE on the Production of Reactive Oxygen Species in AGS and MKN-28 Cells

**Figure 4:**
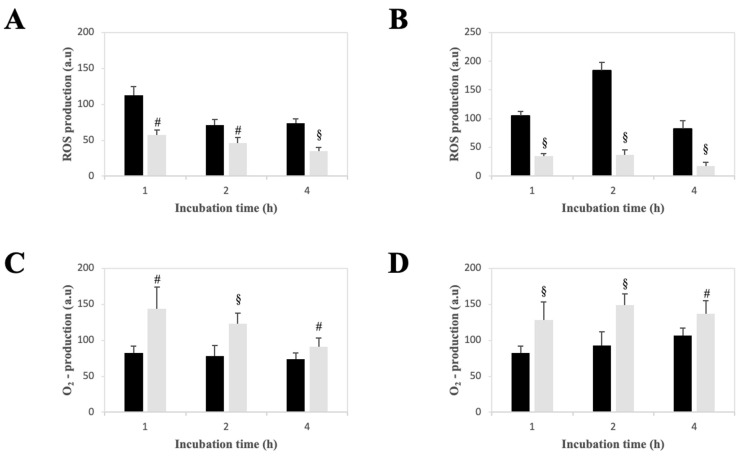
Effect of LPE on the intracellular ROS level. AGS (**A**,**C**) and MKN-28 (**B**,**D**) cells were incubated with PBS, as a vehicle alone (black columns), or with 25 µg/mL LPE (grey columns) for the indicated times. The intracellular ROS level was evaluated through the usage of the fluorescent probe DCFH-DA (**A**,**B**) or DHE (**C**,**D**). Fluorescence intensity was reported as Arbitrary Units (A.U.). Data from triplicate experiments are reported as the mean ± SE. The significance was evaluated with *p* < 0.01 (#) and 0.001 (§) compared to untreated cells.

It is known that polyphenols are involved in the regulation of reactive oxygen species (ROS). Therefore, the intracellular ROS production was measured using the fluorescent probes DCFH-DA, used for the evaluation of hydrogen peroxide and other superoxide-derived ROS, and DHE, a probe that mainly detects the superoxide anions (Figure 4). AGS and MKN-28 cells were treated for 1, 2, and 4 h with PBS, as a vehicle alone, or with 25 µg/mL LPE. Thirty minutes before the end of treatment, cells were incubated with 10 µM DCFH-DA (Figure 4A) or 10 µM DHE (Figure 4B). When using the DCFH-DA probe, LPE caused a significative decrease in fluorescence intensity in both AGS and MKN-28 cells, thus indicating a reduction in the level of hydrogen peroxide and other superoxide-derived ROS. On the contrary, when using the DHE probe, a significative increase in fluorescence intensity was thoroughly observed for both AGS and MKN-28 cells, a finding indicating that LPE caused an increase in superoxide anions. To further investigate the role of LPE in oxidative stress, cells were pre-treated with the antioxidant N-acetylcysteine (NAC). AGS and MKN-28 cells were pre-incubated with 10 mM NAC for 1 h, followed by the addition of LPE (25 µg/mL) for 1, 2, or 4 h. Thirty minutes before the end of the treatment, cells were incubated with 10 µM DCFH-DA. NAC pre-treatment in combination with LPE led to a further and significant reduction in intracellular ROS levels compared with LPE treatment alone in both cell lines (Supplementary Figure S2).

### 2.6. Effect of LPE on the Protein Expression Levels of SOD-1, SOD-2, CAT, and MAO-A in AGS and MKN-28 Cells

**Figure 5:**
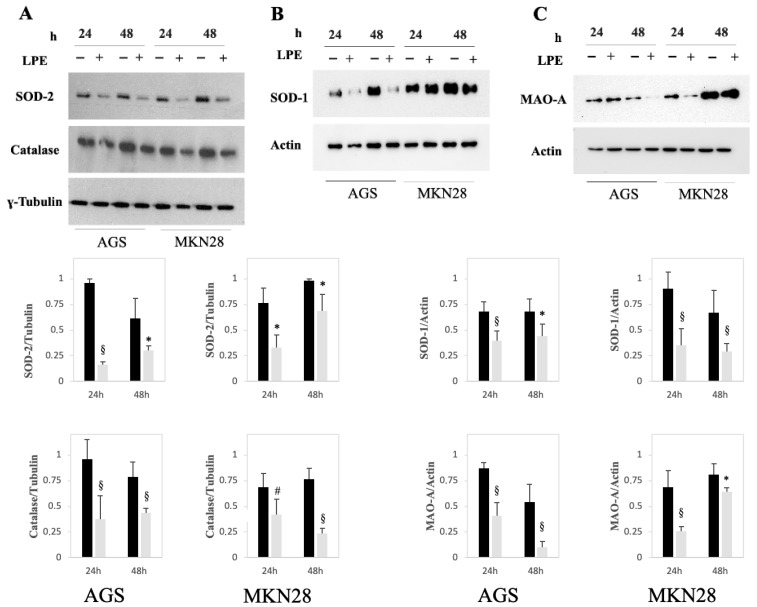
(**A**–**C**) Effect of exposure to LPE on SOD-2, SOD-1, Catalase, and MAO-A protein expression level in AGS and MKN-28 cell lines. Cells were treated with PBS (black bars) or 25 µg/mL LPE (grey bars) for 24 and 48 h. Equal amounts of protein cell lysates (20 μg) were subjected to protein analysis by SDS–PAGE. Western blotting showing protein expression levels in AGS and MKN-28 cells. Each bar represents the mean ± SEM (n = 3). The densitometric evaluation of three independent experiments was reported as the mean ± SE. * *p* < 0.05; # *p* < 0.01; § *p* < 0.001 compared to untreated cells.

To verify the in vitro inhibition by the LPE of key redox enzymes (CAT, SOD-1/2, and MAO-A) in the gastric adenocarcinoma cells [24], we assessed changes in the protein expression of these enzymes after LPE treatment (Figure 5). To this aim, cells were treated with PBS, as a vehicle alone, or with 25 µg/mL LPE for 24 and 48 h and then subjected to Western blotting analysis. The results demonstrated that the exposure to LPE induced in both cell lines a significant reduction in SOD-1, SOD-2, CAT, and MAO-A protein expression levels, compared with those of untreated cells, at both 24 and 48 h of treatment, confirming the inhibitory effect of LPE on these key redox enzymes.

### 2.7. Effect of LPE on SOD Activity in AGS and MKN-28 Cells

**Figure 6:**
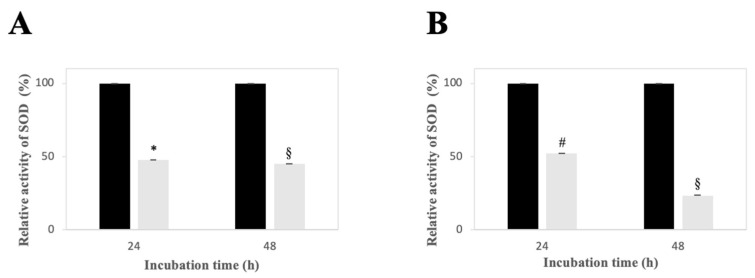
Effect of LPE on SOD activity in AGS and MKN-28 cells. AGS (**A**) and MKN-28 (**B**) cells were incubated with PBS, as a vehicle alone (black columns), or with 25 µg/mL LPE (grey columns) for the indicated times. The relative SOD activity, as a percentage, was measured in the cell lysate using a WST-1–based colorimetric assay. Data from triplicate experiments are reported as the mean ± SE. The significance was evaluated with *p* < 0.05 (*), 0.01 (#), and 0.001 (§) compared to untreated cells.

To further clarify whether the in vitro inhibition of SOD enzymes and the modulation of SOD protein levels observed by Western blotting reflected functional changes, SOD activity was evaluated in lysates from AGS and MKN-28 cells using a WST-1–based colorimetric assay. Cells were treated with PBS as a vehicle control or with 25 µg/mL LPE for 24 and 48 h, and lysates were analyzed spectrophotometrically by evaluating the capacity of endogenous SOD to dismutase superoxide anions generated by a xanthine/xanthine oxidase system, thereby reducing WST-1 formazan formation. As shown in Figure 6, LPE treatment significantly reduced total SOD activity compared with control cells to approximately 50% in AGS cells after 24 and 48 h of treatment and in MKN-28 cells after 24 h. After 48 h of treatment, SOD activity in MKN-28 cells was further decreased to approximately 23%. These results indicate that the downregulation of SOD-1 and SOD-2 protein levels is associated with a functional impairment of SOD enzymatic activity in LPE-treated cells.

### 2.8. Effect of LPE on the Activity of Catalase (CAT)

**Figure 7:**
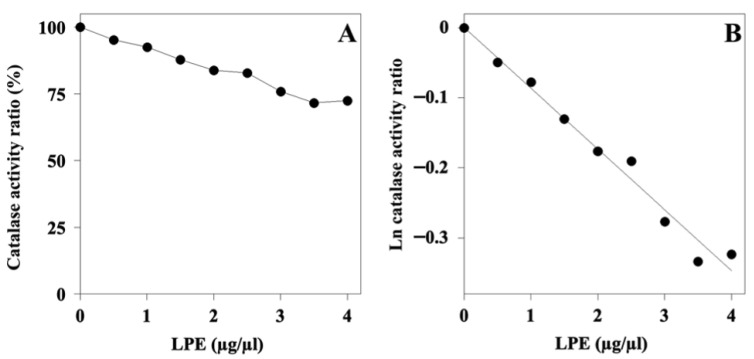
Effect of Lemon Peel Extract on the steady-state activity of bovine liver catalase. (**A**) Ratio of CAT activity was assayed in the presence of indicated LPE concentrations and expressed as a percentage, as indicated in the Methods section. The value obtained in the absence of LPE was 100%. (**B**) Data were also analyzed after a logarithmic transformation of the activity ratio.

It is known that polyphenols exert their antioxidant activity by regulating the intracellular ROS levels and modulating enzymes involved in the antioxidant system. Indeed, in our previous study, LPE inhibited SOD-1 and SOD-2 [24], a finding suggesting a possible reduction in H_2_O_2_, which is produced by SOD. On the other hand, H_2_O_2_ is the main substrate of CAT, and this issue prompted us to investigate whether LPE could affect the activity of this enzyme. The effect of an increasing LPE concentration on the steady-state activity of CAT is illustrated in Figure 7. The extract caused a dose-dependent reduction in the activity (Figure 7A), thus suggesting that the polyphenolic compounds present in the extract were endowed with some inhibitory effect on CAT. After a logarithmic transformation of the activity data (Figure 7B), the calculated IC_50_ value was 0.008 ± 0.003 mg/mL, a value highlighting a moderate inhibition strength by LPE on CAT; when expressed as the molarity of gallic acid equivalent, the IC_50_ of LPE was 47 ± 18 μM.

**Figure 8:**
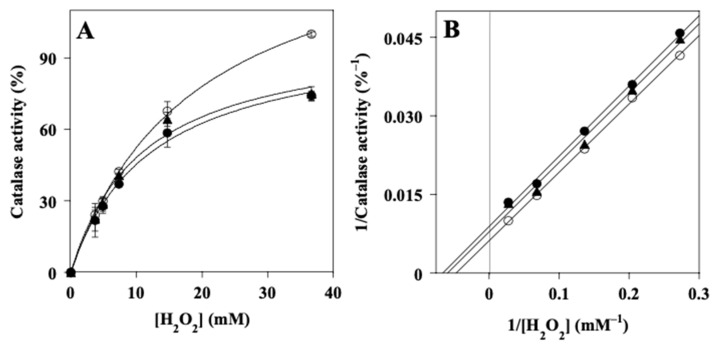
Kinetic analysis of the inhibition of catalase activity by Lemon Peel Extract. Kinetic measurements of catalase activity were realized as reported in Materials and Methods. In particular, H_2_O_2_ ranged between 3.67 and 36.7 mM without (empty circles) or in the presence of 0.0031 mg/mL (triangles) or 0.0062 mg/mL (filled circles) Lemon Peel Extract. CAT activity was expressed as a percentage and 100% was the value obtained with 36.7 mM H_2_O_2_ in the absence of LPE. Data were reported as the mean value +/– SE. (**A**) Michaelis–Menten representation. (**B**) Lineweaver–Burk representation. The correlation coefficient R ranged between 0.992 and 0.999 (**A**) or 0.994 and 0.999 (**B**).

The evaluation of the inhibition mechanism of CAT by LPE was carried out through measurements of the kinetics parameters of CAT activity. To this aim, the data of CAT activity were measured at different hydrogen peroxide concentrations in the absence or in the presence of two fixed concentrations of LPE (Figure 8). The resulting data were analyzed in the typical Michaelis–Menten representation (Figure 8A) and in a Lineweaver–Burk plot (Figure 8B). In particular, the analysis of the parallel straight lines drawn in the Lineweaver–Burk plot could allow for the assignment of an uncompetitive inhibition mechanism of CAT by LPE. However, the proximity of the parallel lines pointing to a moderate inhibition power, and the presence of multiple components in the LPE mixture possibly exhibiting different effects, cannot allow for a clear-cut definition of the inhibition mechanism. From the effect of LPE on the kinetic parameters *K*_M_ and *V*_max_ of CAT activity, as derived from the Michaelis–Menten and Lineweaver–Burk plots, it was possible to obtain the value of the inhibition constant (*K*_i_). The resulting value (*K*_i_ = 0.012 ± 0.002 mg/mL) confirms the moderate inhibition strength of CAT by LPE and represents a more accurate measurement of the inhibition power exerted by the extract, because this parameter is independent of the H_2_O_2_ concentration.

## 3. Discussion

The present study provides new insights into the biological activity of *Citrus limon* peel extract (LPE) and its potential role as an anticancer and redox-modulating agent in human gastric cancer cells. Conventional cancer therapies, including chemotherapy and radiotherapy, are often associated with severe side effects, tumor relapse, and high metastatic potential. Consequently, there is an increasing interest in plant-derived compounds as alternative therapies, particularly polyphenols, due to their lower systemic toxicity and selective anticancer activity. Polyphenols are well known for their antioxidant and chemo-preventive properties; however, depending on the concentration, redox potential, and cellular context, they can also exhibit pro-oxidant behavior. Several dietary polyphenols exhibit a dual antioxidant/pro-oxidant behavior, contributing to their ability to regulate oxidative signaling and inducing apoptosis in cancer cells [29,30,31,32,33]. The LC-MS/MS analysis of LPE led to the identification of its constituents, including ten phenolic acids and eleven flavonoids. Ferulic acid and hesperidin were the most abundant phenolic acid and flavonoid, respectively. The identification of individual components in an extract represents an important tool for understanding their specific effects on metabolic/signaling pathways. However, we cannot exclude that the biological effects of LPE likely arise from synergistic interactions among its constituents [34,35,36]. A previous investigation revealed that LPE inhibited the activity of SOD-1, SOD-2, and other redox enzymes [24]. In this work, we showed that LPE also moderately inhibited catalase (CAT) activity in vitro (IC_50_ = 0.008 ± 0.003 mg/mL; *K*_i_ value = 0.012 ± 0.002 mg/mL). The apparent uncompetitive inhibition mechanism exhibited by LPE could be the result of different effects displayed by the multiple components present in the mixture. Indeed, an alteration of the intracellular hydrogen peroxide turnover affects the overall redox balance, an essential determinant of proliferation and survival in cells. In line with these biochemical findings, LPE treatment significantly affected gastric cancer cell morphology and viability. Phase-contrast microscopy showed morphological alterations of AGS and MKN-28 cells, including cell rounding and detachment, indicative of cytotoxic or pro-apoptotic effects, particularly at higher LPE concentrations and longer exposure times. In addition, no significant morphological changes were observed in BJ-5ta, a normal fibroblast cell line, suggesting a direct cytotoxic effect against tumor cell lines. The MTT assay confirmed these findings, showing a dose- and time-dependent reduction in cell viability in both AGS and MKN-28 lines. These data are consistent with previous studies showing that citrus peel extracts, rich in flavonoids, exert antiproliferative effects by altering redox balance and activating apoptotic pathways [22,23,37,38]. Moreover, the colony formation assay provided evidence that LPE also suppresses the clonogenic potential of gastric cancer cells, a key feature of tumorigenicity. The marked reduction in the number of colonies at LPE concentrations greater than 10–20 μg/mL suggests that LPE compromises the long-term proliferative capacity of these cells. These data reinforce the hypothesis that LPE interferes with fundamental mechanisms of cancer cell survival and replication. An analysis of intracellular ROS production revealed a dual effect of LPE: a significant decrease in hydrogen peroxide-related ROS levels and a concomitant increase in superoxide anion production. This apparent paradox may be explained by the redox-modulating properties of polyphenols, acting as antioxidants or pro-oxidants based on the cellular context [39]. Interestingly, pre-treatment with the antioxidant N-acetylcysteine (NAC) further reduced hydrogen peroxide-related ROS levels compared with LPE alone. This indicates that the effect of lowering the ROS of LPE can be enhanced by classical antioxidants. In our previous study, we demonstrated that LPE inhibits both SOD-1 and SOD-2 in vitro in a concentration-dependent manner [24]. Because SODs catalyze the dismutation of two superoxide anion radicals into H_2_O_2_, their inhibition, together with CAT inhibition, leads to superoxide accumulation, as shown by the enhanced DHE fluorescence, and reduced hydrogen peroxide formation. In addition, LPE polyphenols may directly scavenge H_2_O_2_, contributing to the decreased DCFH-DA fluorescence signal observed. Thus, by inhibiting enzymatic activity and exerting direct antioxidant effects, LPE appears to shift the intracellular ROS balance toward a superoxide-dominant, pro-oxidant state, highlighting a complex and context-dependent redox-regulatory role in gastric cancer cells. Western blotting data also revealed that LPE reduced the protein levels of CAT, SOD-1, SOD-2, and MAO-A in both cell lines after 24 and 48 h of treatment. The rapid downregulation of these enzymes may involve pre- or post-transcriptional mechanisms, including redox-dependent regulation by LPE polyphenols. These enzymes play central roles in maintaining redox equilibrium and mitochondrial integrity; their downregulation can sensitize cancer cells to oxidative stress-induced apoptosis [26]. Although MAO-A activity is primarily involved in neurotransmitter metabolism, it also contributes to ROS generation and has been implicated in tumor development and progression [40,41]. Furthermore, LPE treatment significantly reduced total SOD activity in both AGS and MKN-28 cells, consistent with the decreased SOD-1 and SOD-2 protein levels observed by Western blot. These results suggest that LPE not only reduces enzyme expression but also impairs SOD function, further contributing to superoxide accumulation and a shift toward a pro-oxidant intracellular environment. Taken together, these results suggest that the anticancer activity of LPE involves the complex modulation of cellular redox homeostasis through the inhibition of catalase and other key oxidative enzymes, creating conditions incompatible with cancer cell survival. From a translational perspective, our results highlight lemon peel, an agro-industrial waste product, as a valuable source of bioactive compounds with potential chemo-preventive and therapeutic applications. The identification of specific active constituents within LPE, and the elucidation of their molecular targets, will be crucial to further defining its mechanism of action and potential synergistic effects with conventional anticancer agents.

## 4. Materials and Methods

### 4.1. Materials

The AGS, MKN-28, and BJ-5ta cell lines were from the American Type Culture Collection (Manassas, VA, USA). Dulbecco’s modified Eagle medium (DMEM) and 10% heat-inactivated fetal bovine serum (FBS) were from Microgem Laboratory Research (Milan, Italy). Bovine liver catalase (CAT, 2000–5000 U/mg protein), gallic acid, L-glutamine, penicillin G, streptomycin, 3-(4,5-dimethylthiazol-2-yl)-2,5-biphenyltetrazolium bromide (MTT), crystal violet, N-acetyl- L-cysteine (NAC), and the fluorescent probes dihydroethidium (DHE) and 2′,7′-dichlorofluorescein diacetate (DCFH-DA) were purchased from Sigma-Aldrich (St. Louis, MO, USA). A protease inhibitor cocktail was obtained from Roche Diagnostics S.p.A. (Monza, Italy). Methanol, formic acid, and quinaldic acid were LC-MS grade and purchased from Sigma Aldrich (Darmstadt, Germany). The Phenolic Acids and Alcohols Standard Mixture-V2 and Flavonoids Standard Mixture-V2 were obtained from MetaSci library (https://www.metasci.ca/, accessed on 30 December 2025). These substances were used for peak identification, Multiple Reaction Monitoring (MRM) method development, and calibration curves. All other reagents were of analytical grade.

### 4.2. Methods

#### 4.2.1. Preparation of Lemon Peel Extract (LPE)

*Citrus limon* peels, derived from vegetable waste for liquor production, were kindly supplied by Villa Massa (Piano di Sorrento, NA, Italy). LPE was obtained as previously described [17,22,23,24]. The lyophilized material obtained was resuspended in phosphate-buffered saline (PBS) and stored at −20 °C until it was used.

#### 4.2.2. Determination of Total Phenolic Acids Content

The total content of phenolic acids was determined according to the adapted Folin–Ciocalteu colorimetric method [42] and the results were expressed as the molarity of gallic acid equivalent (GAE) per g of dry sample. The chemical composition of LPE previously reported [17] was confirmed by reverse-phase (RP)–high-performance liquid chromatography (HPLC) [24].

#### 4.2.3. LC-MS/MS Analysis of Phenolic Acids and Flavonoids

The LC-MS/MS system used for the analysis of the extracts included a UHPLC (Nexera Series LC-40, Shimadzu, Kyoto, Japan) coupled to a triple quadrupole/linear ion trap tandem mass spectrometer (QTRAP 4500, AB Sciex, Framingham, MA, USA) that was equipped with a Turbo V ion source. Instrument control, data acquisition, and processing were achieved by the associated Analyst 1.6 and Multiquant 3.0 software. The quantification of phenolic acids and flavonoids and the validation of chromatographic methods were conducted in the same conditions as previously reported [27].

#### 4.2.4. Cell Cultures

The human gastric adenocarcinoma AGS and MKN-28 cell lines and the hTERT-immortalized fibroblast BJ-5ta cell line were cultured in DMEM supplemented with 10% heat-inactivated FBS, 2 mM L-glutamine, 100 IU/mL penicillin G, and 100 μg/mL streptomycin, in a humidified incubator at 37 °C under a 5% CO_2_ atmosphere. Cells were split and seeded in plates (75 cm^2^) every 2 days and used for assays during the exponential phase of growth. Cell treatments were always carried out 24 h after plating.

#### 4.2.5. Morphological Analysis

Cell morphology observations were carried out as previously reported [43]. Briefly, the cells were seeded subconfluently onto a six-well plate, treated with different LPE concentrations, and then observed for 24 and 48 h using a phase-contrast microscope from Carl Zeiss (Jena, Germany), HBO 50/ac model; images were acquired with a digital video camera (Panasonic Lumix DC-FZ82 Bridge, Panasonic, Kadoma City, Japan) connected to the microscope.

#### 4.2.6. Cell Viability Assay

The cell viability was evaluated as mitochondrial metabolic activity using the MTT assay, as previously reported [44]. Briefly, cells were seeded into 96-well microplates (1 × 10^4^ cells/well) and, after 24 h of incubation, treated with different concentrations of LPE (2.5–40 µg/mL) or with PBS as the control vehicle. After 24 and 48 h of treatment, 10 μL of the MTT solution (5 mg/mL) was added to each well in the dark, and the plates were incubated for 3 h at 37 °C under a 5% CO_2_ atmosphere. At the end of incubation, the culture medium was removed, and 100 μL of 0.1 N hydrochloric acid in isopropanol was added to each well to solubilize the formazan crystals. Finally, the absorbance was measured at a wavelength of 570 nm using a BioTek Synergy LX microplate reader (Agilent, Santa Clara, CA, USA). The cell viability was expressed as a percentage relative to the untreated cells set as 100%.

#### 4.2.7. Colony Formation Assay

The colony-forming assay was performed as previously described [45]. Briefly, cells were seeded in six-well plates at a density of 4 × 10^2^ cells/well. After 2/3 days, the cells were treated with PBS as a control vehicle or different LPE concentrations (5–40 µg/mL) and incubated for additional 7 days at 37° C. Then, the colonies were stained with 1% (*w*/*v*) crystal violet in 50% (*v*/*v*) ethanol for 1 h at room temperature. The cells were photographed with a digital camera (Panasonic Lumix DC-FZ82 Bridge); the number of colonies was counted using ImageJ 1.54d software.

#### 4.2.8. Measurement of Intracellular ROS Content

The intracellular ROS levels were detected using the oxidation-sensitive fluorescent probes DHE and DCFH-DA [46,47]. Briefly, cells were seeded into six-well plates (3 × 10^5^ cells/well) and treated with LPE (25 µg/mL) or PBS, as a vehicle control, for different times. DHE or DCFH-DA was added in the dark at a final concentration of 10 µM, 30 min before the end of each incubation. Then, cells were collected, washed with PBS, and resuspended in PBS for the fluorometric analysis. The effect of LPE on ROS production was also evaluated after the pre-treatment of cells with 10 mM NAC for 1 h. Measurements were performed using a Cary Eclipse fluorescence spectrophotometer (Varian, Palo Alto, CA, USA). Excitation and emission wavelengths were set at 358/461 nm for DHE and 485/538 nm for DCFH-DA, respectively, with both excitation and emission slits widths set at 10 nm.

#### 4.2.9. Total Cell Lysates for Western Blotting Analysis

To obtain total protein extracts from AGS and MKN-28, cells were seeded into six-well plates at a density of 3 × 10^5^ cells per plate and incubated for 24 h at 37 °C. The cells were then treated with LPE (25 µg/mL) or PBS, as a control vehicle. After 24 h of treatment, cells were harvested, washed with PBS, and then lysed in an ice-cold modified radioimmunoprecipitation assay (RIPA) buffer (50 mM Tris-HCl, pH 7.4, 150 mM NaCl, 1% Nonidet P-40, 0.25% sodium deoxycholate, 1 mM Na_3_VO_4_, and 1 mM NaF), supplemented with protease inhibitors, and incubated for 30 min on ice. The supernatant, obtained after centrifugation at 13,200× *g* for 30 min at 4 °C, constituted the total protein extract. The protein concentration was determined using the Bradford method with bovine serum albumin (BSA) as the standard. Equal amounts of total protein extracts were used for Western blotting analysis. Briefly, protein samples were dissolved in SDS-reducing loading buffer and separated by sodium dodecylsulfate polyacrylamide gel electrophoresis (SDS/PAGE). The proteins were then transferred to Immobilon P membrane (Millipore, St. Louis, MO, USA). The membrane was then incubated with the specific primary antibody at 4 °C overnight, followed by incubation with the secondary antibody at room temperature for 1 h. The primary antibodies used were SOD-1 (FL-154) Rabbit polyclonal antibody [sc-11407] (1:1000 dilution) (Santa Cruz Biotechnology, Dallas, TX, USA); Anti-Mn-SOD Rabbit polyclonal antibody [06-984] (1:1000 dilution) (Millipore, St. Louis, MO, USA); Anti-Catalase Rabbit polyclonal antibody [219010-M] (1:3000 dilution) (Calbiochem^®^, San Diego, CA, USA); Recombinant Anti-Monoamine Oxidase A/MAO-A antibody [EPR7101] (1:500 dilution) (Abcam, Cambridge, UK); Bcl-xL (H-5) Mouse monoclonal antibody [sc-8392] (1:200 dilution) (Santa Cruz Biotechnology, Dallas, TX, USA); [KO Validated] Bax Rabbit monoclonal antibody [A19684] (1:1000 dilution) (ABclonal, Düsseldorf, DE); Anti-gamma Tubulin mouse monoclonal antibody [T6657] (1:5000 dilution) (Sigma-Aldrich, St. Louis, MO, USA); and beta-Actin mouse monoclonal antibody (C4) (1:1000 dilution) [sc-47778] (Santa Cruz Biotechnology, Dallas, TX, USA). Membranes were then analyzed using an enhanced chemiluminescence reaction with WesternBright ECL (Advansta, San Jose, CA, USA) according to the manufacturer’s instructions. Signals were visualized using a Chemidoc MP Imaging System (Bio-Rad, Hercules, CA, USA).

#### 4.2.10. SOD Activity Assay

SOD activity in AGS and MKN-28 cells was measured using the SOD Assay Kit-WST (Dojindo Laboratories, Kumamoto, Japan), following the manufacturer’s instructions. Cells were seeded into a 75 cm^2^ plate at a density of 2 × 10^6^ cells and incubated for 24 h at 37 °C. The cells were then treated with PBS, as a vehicle control, or with 25 µg/mL LPE for 24 and 48 h. After incubation times, the cells were harvested, washed, and lysed. The protein concentration was determined by the Bradford assay, and equal amounts of lysate (50 µg) were used for the assay. In total, 20 µL of cell lysate was added to each well of a 96-well plate, followed by 200 µL of WST working solution and 20 µL of the enzyme solution containing xanthine and xanthine oxidase, which generates superoxide anions. Plates were incubated at 37 °C for 20–30 min, and the absorbance at 450 nm was measured using a BioTek Synergy LX microplate reader (Agilent, Santa Clara, CA, USA). SOD activity was calculated based on the inhibition of WST-1 formazan formation relative to control wells and expressed as a percentage.

#### 4.2.11. Biochemical Methods

The activity of CAT was measured with a spectrophotometric method, using a Cary 100 UV-Vis Spectrophotometer (Agilent Technologies, Milan, Italy) and setting the wavelength at 240 nm, essentially as previously described [48]. In the steady-state CAT assay, the 1 mL final volume of a 50 mM potassium phosphate buffer, pH 7.0 (buffer A), contained 1175 mM hydrogen peroxide and different concentrations of the various LPE extracts. The reaction started at 25 °C with the addition of 1 U/mL of CAT. The absorbance decrease was kinetically recorded up to 30 s to evaluate the linear part of the kinetics. The effect of LPE was evaluated in a dose-dependent profile, reporting the ratio of CAT activity measured in the presence of the extract over that measured in its absence, vs. the concentration of the extract, evaluated as the molarity of a gallic acid equivalent [42]. A 1 μM gallic acid equivalent corresponded to 0.17 μg/mL. The LPE concentration causing a 50% reduction in CAT activity (IC_50_) was evaluated from semilogarithmic plots of the CAT activity data. The inhibition power and mechanism exerted by LPE were evaluated from kinetic measurements of CAT activity, performed in the absence or in the presence of two fixed concentrations of LPE. To this aim, the substrate concentration in the reaction mixtures ranged between 3.67- and 36.7 mM hydrogen peroxide, while the other experimental conditions remained those of the steady-state assay. The *K*_M_ for the substrate and *V*_max_ of the reaction were derived from Michaelis–Menten and Lineweaver–Burk plots, as previously reported [48], and the putative type of the inhibition mechanism was tentatively assessed based on the effect of LPE on *K*_M_ and *V*_max_. An uncompetitive inhibition mechanism was suggested from the concomitant decrease in *K*_M_ and *V*_max_ values. The inhibition constant (*K*_i_) was obtained using the equations expected for an uncompetitive mechanism:

*K*_i_ = *V*′_max_ × [I]/(*V*_max_ − *V*′_max_) or *K*_i_ = *K*′_M_ × [I]/(*K*_M_ − *K*′_M_)

where *V*′_max_ and *K*′_M_ represent the *V*_max_ and *K*_M_ measured in the presence of the concentration [I] of LPE.

### 4.3. Statistical Analysis

Data are reported as the mean ± standard error (SE) of at least three independent experiments performed in triplicate. The statistical significance of differences among groups was evaluated using ANOVA, with the Bonferroni correction as a post hoc test or the Student *t*-test, where appropriate. Significant differences were considered as follows: * *p* < 0.05; # *p* < 0.01; § *p* < 0.001.

## 5. Conclusions

In conclusion, LPE exhibits significant cytotoxic, redox-modulating, and enzyme-inhibitory activities in human gastric cancer cells. Treatment with LPE substantially decreased cell viability and clonogenic potential while shifting the intracellular ROS balance toward a superoxide-dominant, pro-oxidant state. These effects were accompanied by the downregulation of key antioxidant enzymes, indicating a complex modulation of redox homeostasis that compromises cancer cell survival. Overall, these findings highlight the potential of LPE as a natural source of bioactive compounds for developing novel strategies in gastric cancer prevention or therapy. Future studies on the effects produced by the individual polyphenolic components identified could clarify their molecular mechanisms and assess the safety and efficacy of LPE in preclinical cancer models. Moreover, the combination of these polyphenols with existing FDA-approved drugs could have great potential in enhancing their clinical effectiveness.

## Figures and Tables

**Table 1:** Phenolic acids and flavonoids detected in *Citrus limon* peel extracts (LPE).

Compound	Type	µg/mL
Chlorogenic acid	Polyphenol	<LOQ *
*p*-Coumaric acid	“	4.36 ± 1.50
Ferulic acid	“	7.70 ± 3.10
Sinapic acid	“	<LOQ
4-Hydroxybenzoic acid	“	1.42 ± 0.17
Gentisic acid	“	<LOQ
Salicylic acid	“	<LOQ
*trans*-2-Hydroxycinnamic acid	“	2.69 ± 0.56
3-Methoxyhydrocinnamic acid	“	<LOD **
Caffeic acid phenethyl ester	“	<LOQ
Kaempferol	Flavonoid	2.05 ± 0.84
Myricetin	“	9.63 ± 0.01
Diosmetin	“	0.13 ± 0.11
Morin	“	<LOQ
Chrysin	“	<LOQ
(+/−)-Naringenin	“	0.70 ± 0.05
Baicalin	“	<LOD
Hesperidin	“	202.06 ± 14.5
Rutin Hydrate	“	48.49 ± 1.14
Daidezin	“	<LOQ
Biochanin A	“	<LOQ

* Below the limit of quantification (LOQ); ** Below the limit of detection (LOD).

## Data Availability

The original contributions presented in this study are included in the article/Supplementary Material. Further inquiries can be directed to the corresponding author.

## Supplementary Materials

The following supporting information can be downloaded at: https://www.mdpi.com/article/10.3390/ijms27020598/s1.
