# Supplementary material for: Citrus limon Peel Extract Modulates Redox Enzymes and Induces Cytotoxicity in Human Gastric Cancer Cells
Source: Int J Mol Sci. 2026 Jan 7;27(2):598. doi: 10.3390/ijms27020598 (PMC12841246; doi:10.3390/ijms27020598)

Supplementary material

Citrus limon peel extract modulates redox enzymes and induces cytotoxicity in human gastric cancer cells

Rosarita Nasso[1], Rosario Rullo[2], Antonio D'Errico[1], Pierluigi Reveglia[3], Lucia Lecce[3], Annarita Poli[4], Paola Di Donato[5], Gaetano Corso[3], Emmanuele De Vendittis[6], Rosaria Arcone[1], Mariorosario Masullo[1,*]

[1] Department of Medical, Movement and Well-Being Sciences, Via Medina, 40, 80133 Napoli, Italy; rosaritanasso@gmail.com (R.N.); antonio.derrico002@studenti.uniparthenope.it (A.D.); rosaria.arcone@uniparthenope.it (R.A.); mario.masullo@uniparthenope.it (M.M.)

[2] Institute for the Animal Production Systems in the Mediterranean Environment, National Research Council, Piazzale Enrico Fermi 1, 80055 Portici, Italy; rosario.rullo@cnr.it (R.R.)

[3] Department of Clinical and Experimental Medicine, University of Foggia, Viale Pinto, 1, 71122 Foggia, Italy; pierluigi.reveglia@unifg.it (P.R.); lucia.lecce@unifg.it (L.L.), gaetano.corso@unifg.it (G.C.)

[4] Institute of Biomolecular Chemistry, National Research Council, Via Campi Flegrei 34, 80078 Pozzuoli (NA), Italy; annarita.poli@cnr.it (A.P.)

[5] Department of Science and Technologies, University of Naples "Parthenope", Centro Direzionale Isola C4, 80143 Napoli, Italy; paola.didonato@uniparthenope.it (P.D.D.)

[6] Department of Molecular Medicine and Medical Biotechnologies, University of Naples Federico II, Via S. Pansini 5, 80131 Napoli, Italy; devendit@unina.it (E.D.V.)

* Correspondence mario.masullo@uniparthenope.it; Tel.: +39.081.5474676

Figure S1 – Effect of LPE on the cell morphology of BJ-5ta immortalized fibroblast cell lines.

Figure S2 – Effect of LPE and NAC on intracellular ROS levels in AGS and MKN-28 cells.

Figure S1 – Effect of LPE on the cell morphology of BJ-5ta immortalized fibroblast cell lines. BJ-5ta cells were treated with vehicle alone (PBS) or 5, 10, 20, 40 µg/ml LPE for 24 and 48 h. Images are representative of three independent experiments. Magnification x10. (see Material and Methods section 4.2.5.).

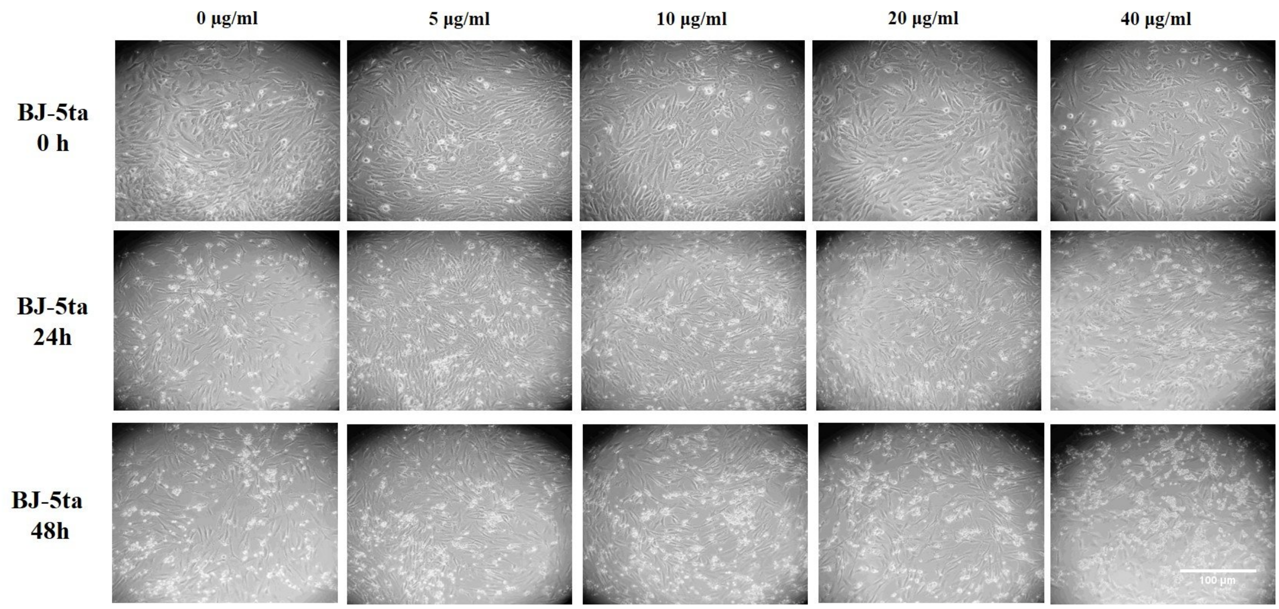

Figure S2 – Effect of LPE and NAC on intracellular ROS levels in AGS and MKN-28 cells. Intracellular ROS levels were measured by DCFH-DA fluorescence in AGS (**A**) and MKN-28 (**B**) cells. Cells were treated for the indicated times with PBS, as vehicle alone (black columns), 25 µg/mL LPE (grey columns), 10 mM NAC (striped colunms), or NAC + LPE (dotted colunms). NAC was added 1 hour before LPE, and DCFH-DA was added 30 minutes before the end of treatment. Data from triplicate experiments are reported as mean ± SE. The significance was evaluated with $p < 0.01$ (#), 0.001 (§) compared to untreated cells. (see Material and Methods sections 4.2.8.).

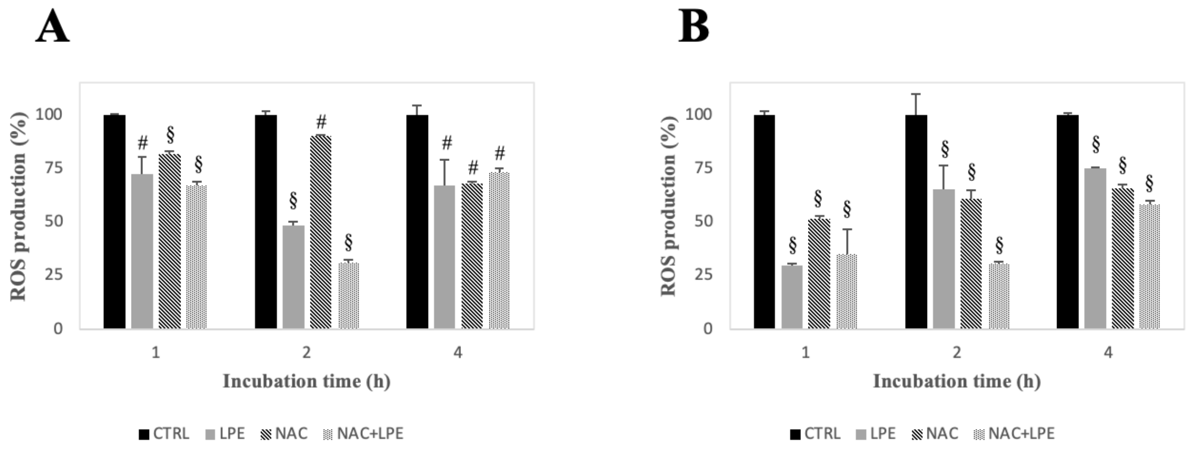

Supplement: Supplementary file 1 [file ijms-27-00598-s001.zip › ijms-4043390-supplementary.pdf]
